# Routine exercise testing could not predict T‐wave oversensing in a patient after a subcutaneous implantable cardioverter‐defibrillator implant

**DOI:** 10.1002/ccr3.1345

**Published:** 2017-12-22

**Authors:** Shozo Konishi, Hitoshi Minamiguchi, Kentaro Ozu, Hiroya Mizuno, Shungo Hikoso, Osamu Yamaguchi, Yasushi Sakata

**Affiliations:** ^1^ Department of Cardiovascular Medicine Osaka Graduate School of Medicine Suita Japan

**Keywords:** Exercise test, subcutaneous implantable cardioverter‐defibrillator, T‐wave oversensing

## Abstract

Subcutaneous implantable cardioverter‐defibrillators (S‐ICDs) are susceptible to T‐wave oversensing (TWOS) caused by high rate‐dependent QRS–T morphology changes. We experienced an inappropriate S‐ICD shock due to TWOS, which could not be predicted by routine exercise testing. A newly available high‐pass filter might be effective for avoiding this.

## Introduction

The subcutaneous implantable cardioverter‐defibrillator (S‐ICD) is a novel treatment modality for preventing sudden cardiac death that does not require a lead implantation in the heart; however, it still is associated with a 13% rate of inappropriate shocks (IASs) over 3 years based on the IDE Study and EFFORTLESS Registry 1, 2, 3. The majority of IASs are due to T‐wave oversensing (TWOS), which has been reduced by exercise testing 4, 5. Here, we report the case of a patient who experienced an IAS due to TWOS, which a prior exercise test could not predict.

## Case Report

**Figure 1:**
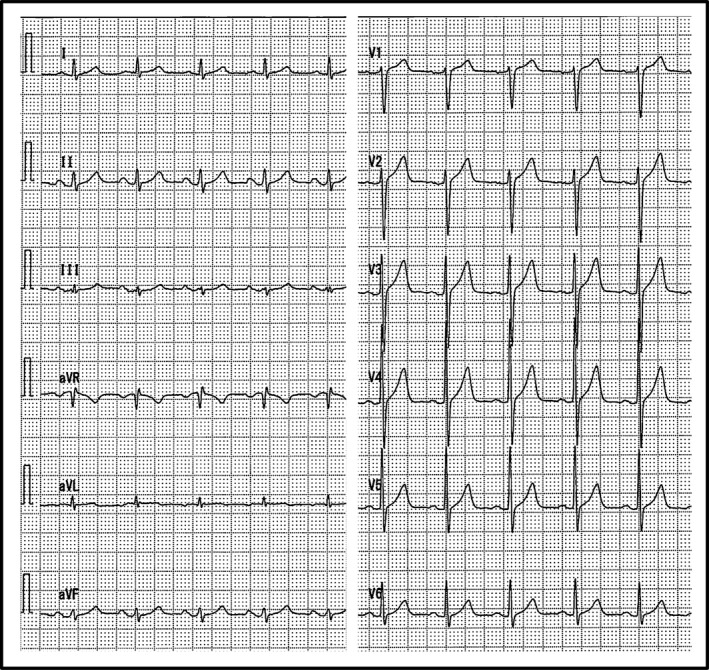
12‐lead electrocardiogram at rest. A 12‐lead electrocardiogram revealed sinus rhythm without any evidence of an old myocardial infarction, Brugada syndrome, early repolarization syndrome, or QT‐interval abnormalities.

**Figure 2:**
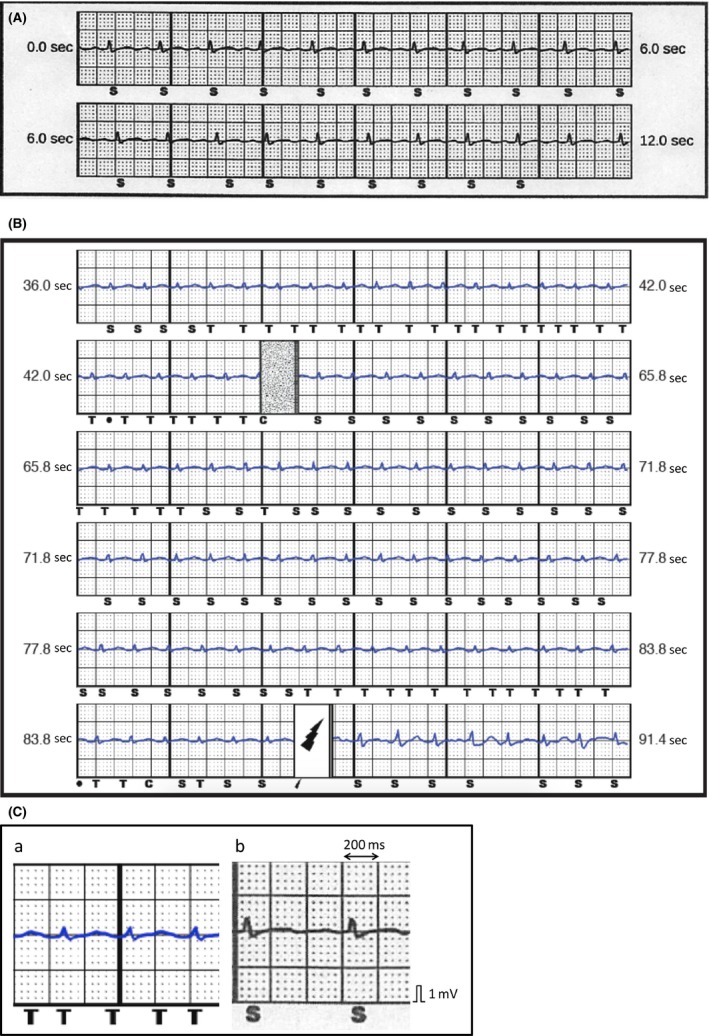
(A) Subcutaneous implantable cardioverter‐defibrillator recording of a secondary vector during a prior scheduled visit. T‐wave sensing was sometimes observed; however, the intrinsic subcutaneous implantable cardioverter‐defibrillator unique algorithm successfully avoided any “double counting” in normal sinus rhythm. (B) Subcutaneous implantable cardioverter‐defibrillator recording of a secondary vector during an inappropriate shock. Intermittent T‐wave oversensing and “double counting” were observed prior to an inappropriate shock. (C) Magnified QRS morphology recorded by the subcutaneous implantable cardioverter‐defibrillator. A more decreased QRS amplitude and increased T‐wave amplitude were observed during the inappropriate shock (C‐a), as compared to that during the prior scheduled visit (C‐b).

A 61‐year‐old ventricular fibrillation (VF) survivor underwent a dual chamber transvenous implantable cardioverter‐defibrillator (TV‐ICD) implantation for secondary prevention of sudden cardiac death. He did not have any syncopal episodes and had no family history of sudden cardiac death. A 12‐lead electrocardiogram (ECG) revealed sinus rhythm without any evidence of an old myocardial infarction, Brugada syndrome, early repolarization syndrome, or QT‐interval abnormalities (Fig. 1). An echocardiogram revealed a normal left ventricular ejection fraction, without any local asynergy or valvular heart disease. There was no sign of coronary artery or structural heart disease. He was diagnosed with idiopathic VF. After 4 years, he experienced a TV‐ICD pocket infection caused by *S. aureus* and was referred to our hospital. The whole TV‐ICD system was successfully extracted using an excimer laser sheath under general anesthesia. After the TV‐ICD extraction, from subcutaneous cardiac defibrillator to subcutaneous implantable cardioverter‐defibrillator (S‐ICD) was chosen, because he had no indication for pacing and pre‐ECG screening showed that two of the three vectors were recognized as acceptable. The S‐ICD was successfully implanted in the standard position using a standard fashion. The alternate vector was selected as the optimal sensing vector based on an automatic S‐ICD analysis during rest, and the device was programed with a conditional zone of over 200 beats per minute (bpm) and a shock zone of over 230 bpm. One month after discharge, on his scheduled visit, a secondary vector was selected as the optimal sensing vector based on the automatic S‐ICD analysis. Then, he underwent exercise testing as a routine test after the implantation. His heart rate increased from 90 bpm to a maximum of 140 bpm, at which point he could not continue exercise any further due to leg fatigue. No IAS was observed during the exercise test under the secondary vector (Fig. 2A).

However, 10 months after the procedure, he experienced an IAS when he was in a dehydrated state because of a high fever. The stored QRS morphology during the IAS differed from that during the prior scheduled visit (Fig. 2B and C). The QRS duration was wider and the QRS amplitude was decreased; however, on the other hand, the T‐wave amplitude had increased during the IAS event. Sinus tachycardia might have caused a heart rate‐dependent ventricular conduction disturbance, which could have led to intermittent TWOS. The secondary vector was still acceptable as the optimal sensing vector, based on the automatic reanalysis by the S‐ICD at that time. The patient underwent exercise testing with maximum effort for the second time; however, the achieved maximum heart rate of 140 bpm did not reproduce any ventricular conduction disturbance. A newly available high‐pass filter (SMART PASS filter) software upgrade was applied to avoid any further TWOS. He has experienced no IASs for 8 months after the use of the high‐pass filter.

## Discussion

The S‐ICD is a recently introduced technology for the prevention of sudden cardiac death with clinical trial‐supported efficacy 1, 2, 3. The advantage of the S‐ICD is that it is entirely subcutaneous, avoiding the need for transvenous leads and their associated complications. This device should be a promising tool for TV‐ICD infection survivors when pacing therapy for bradycardia support, cardiac resynchronization, or antitachycardia pacing is not necessary. On the other hand, as the leads are extracardiac, the sensing and programing differ from that of the TV‐ICD, posing new challenges. The 360‐day rate of IASs observed in the EFFORTLESS S‐ICD Registry was 7% 5. Supraventricular tachycardias account for the majority of IASs associated with TV‐ICDs, whereas oversensing, especially TWOS, is the primary cause of IASs associated with S‐ICDs 2, 4, 5, 6, 7. Because the S‐ICD relies on “far‐field” electrograms (resembling a surface electrocardiogram), it may be more sensitive to QRS–T wave morphology changes than the local “near‐field” ventricular electrograms of the TV‐ICDs, rendering the S‐ICD more prone to TWOS. Further, the S‐ICD has a fixed sensing algorithm that can only be adjusted by changing the therapy zone frequency or by changing the sensing vector and stored template.

This case highlights the difficulties in predicting TWOS due to the dynamic changes in the QRS–T wave morphology. Exercise testing has also been proposed as an effective test post‐S‐ICD implantation to guide the vector selection for reducing TWOS^4^; however, in this case, even repeated exercise tests could not reproduce any ventricular conduction disturbance. This was probably because the heart rate during the IAS event was higher than that achieved during the exercise tests with maximum effort. Although the maximum heart rate was 140 bpm during the exercise tests, the heart rate during the IAS was over 150 bpm and after that, it gradually came down as his fever came down. The tachycardia during the IAS was not likely an atrial tachyarrhythmia but was most likely sinus tachycardia. With the S‐ICD, when a ventricular conduction disturbance arises, the QRS–T wave morphology recorded by the device may also change. In such a situation, the R/T wave ratio may decrease to a threshold where the T wave is above the sensing decay curve following the R wave detection and is therefore counted by the device's detection algorithm. Atrial high rate pacing also may not mimic physiological sinus tachycardia because the refractory period of the conduction system or ventricular myocardium is not modulated by excited sympathetic nervous activity. In Brugada syndrome, an exercise test cannot always predict TWOS and one possible solution to avoid TWOS might be drug challenging testing 8. However, this case was diagnosed with idiopathic VF, not Brugada syndrome.

Another possible solution to avoid TWOS would be the newly available high‐pass filter 9. In our case, the device was not equipped with the high‐pass filter and was not available at implant. Most recently, it has been reported that the high‐pass filter raises the QRS/T ratio much higher, indicating a lesser susceptibility to oversensing. We think that the high‐pass filter would be one of the effective solutions to avoid TWOS even when a ventricular conduction disturbance occurs.

## Conclusion

We report a case of an S‐ICD IAS due to a rate‐dependent QRS morphology change. We should recognize that exercise testing does not always work well for predicting dynamic QRS morphology changes. The high‐pass filter could be one of the solutions to avoid TWOS associated with S‐ICD systems.

## Authorship

SK and HM: performed medical practice and drafted the article. KO, HM: performed medical practice. SH, OY, YS: made critical revision.

## Conflict of Interest

None declared.
